# Supplementary material for: The discovery of novel noncoding RNAs in 50 bacterial genomes
Source: Nucleic Acids Res. 2024 Apr 22;52(9):5152–65. doi: 10.1093/nar/gkae248 (PMC11109978; doi:10.1093/nar/gkae248)
Supplement: gkae248_Supplemental_Files [file gkae248_supplemental_files.zip › Narunsky 50 Genomes RNA Motifs Files.zip!/Publication/59-G_aurantiaca/Figures/LRC-59-1-NC_012489_1_IGR_9_995869_996065.sto.pdf]

LRC-59-1-NC_012489_1_IGR_9_995869_996065.sto.r2r

5′-○○○○○○○○R●C●●A●CGA●●R●A●Y●C●●AAGYCAGUCCA●CCUA●CACCUAC●●C●UAUCACCYA●YACCAAGCYCCCUAARACC●RYG●C●●C●R●●●○○○○○○○○○○○○○○○○○○○○○
